# Rewiring the T cell-suppressive cytokine landscape of the tumor microenvironment: a new frontier for precision anti-cancer therapy

**DOI:** 10.3389/fimmu.2024.1418527

**Published:** 2024-08-30

**Authors:** Ludovica Lopresti, Vanessa Tatangelo, Cosima T. Baldari, Laura Patrussi

**Affiliations:** Department of Life Sciences, University of Siena, Siena, Italy

**Keywords:** tumor microenvironment, cytokine, interleukin, T cell suppression, cytotoxic T lymphocyte, TIL, immune checkpoint

## Abstract

T lymphocytes that infiltrate the tumor microenvironment (TME) often fail to function as effective anti-cancer agents. Within the TME, cell-to-cell inhibitory interactions play significant roles in dampening their anti-tumor activities. Recent studies have revealed that soluble factors released in the TME by immune and non-immune cells, as well as by tumor cells themselves, contribute to the exacerbation of T cell exhaustion. Our understanding of the cytokine landscape of the TME, their interrelationships, and their impact on cancer development is still at its early stages. In this review, we aim to shed light on Interleukin (IL) -6, IL-9, and IL-10, a small group of JAK/STAT signaling-dependent cytokines harboring T cell-suppressive effects in the TME and summarize their mechanisms of action. Additionally, we will explore how advancements in scientific research can help us overcoming the obstacles posed by cytokines that suppress T cells in tumors, with the ultimate objective of stimulating further investigations for the development of novel therapeutic strategies to counteract their tumor-promoting activities.

## 1 Introduction

The 3E theory - “Elimination, Equilibrium, Escape” – was first proposed in 2004 by Dunn and colleagues (1). According to this model, cells and molecules of the immune system destroy pre-malignant and malignant cells as they emerge (Elimination). Cancer cells that get over the elimination phase undergo a series of genomic and epigenomic changes and acquire the ability to survive (Equilibrium), becoming resilient to immune system controls (Escape). Failure of immune cells to control neoplastic cells represents therefore the foundation of cancer progression (2).

T lymphocytes are the host’s most potent “immune weapons” against cancer. Their effector subtypes, helper T cells (TH) and cytotoxic T cells (CTLs), fight enemies either indirectly, by secreting a plethora of soluble factors that activate other immune cells, or directly, by destroying tumor cells. These mechanisms mainly rely on the ability of T Cell Receptors (TCRs) to recognize cognate foreign antigens loaded on major histocompatibility complex (MHC) molecules. However, efficient immune responses also require a wide range of safety controls, that include inhibitory proteins called “immune checkpoints”, expressed on the surface of T cells where they contribute to ensure self-tolerance (3). In recent years it has become increasingly clear that inhibitory pathways elicited by immune checkpoints are double-edged swords, as they become aberrantly activated in some pathological contexts, including acute or persistent chronic infections (4, 5) and cancer (6), thereby suppressing the effector functions of T lymphocytes and hindering the host’s anti-tumor responses (6).

A vast spectrum of factors that harness T lymphocyte anti-cancer activities is progressively coming to prominence. Nutrient deficiency, hypoxia, dysregulated purinergic and bioenergetic signaling, increased amount of extracellular ATP, adenosine, and potassium ions, generated or released by dying and/or necrotic tumor cells and by neighboring cells in the tumor microenvironment (TME), promote tumor immune escape (2). Cytokines, the quintessential soluble mediators of cell-to-cell communication, rightfully belong to this heterogeneous list of extrinsic pro-tumoral factors. Indeed, besides their key roles in immune cell activation, recruitment and differentiation, which make them the most important mediators of immune system functions, they also exert tumor-promoting activities (7). A subgroup of cytokines released in the TME by immune and non-immune cells have been found to suppress the effector functions of T lymphocytes (8). Interestingly, while some of them primarily act on tumor cells, which subsequently acquire the ability to impair the anti-tumoral responses of T lymphocytes, a small group of cytokines directly act on T lymphocytes to hamper their activities, thereby making T lymphocytes unable to fight against tumor cells. Here we summarize the most recent findings concerning the small group of JAK/STAT signaling-dependent cytokines Interleukin (IL) -6, IL-9, and IL-10, which have been recently implicated in targeting T lymphocytes and hampering their anti-tumoral functions, briefly summarizing their mechanisms of action. We moreover discuss potential strategies to revitalize T lymphocytes in the TME and augment the clinical benefit of immunotherapy.

## 2 Positive and negative regulators of antigen receptor signaling in T cells

Recognition of MHC-loaded foreign antigens by the TCR is the first step towards T cell activation. However, the TCR is not the sole molecule involved in the activation of this complex machinery. A wide number of co-receptors, co-stimulatory receptors and accessory molecules coordinately concur to generate a polarized structure at the interface between T cells and antigen-presenting cell (APC), known as the immune synapse (IS). IS formation consists of the timely and precise reorganization of surface receptors and intracellular molecules that are involved in antigen recognition and adhesion to APC. Three specialized concentric functional domains can be found in the mature IS, the central supramolecular activation cluster (cSMAC), where TCRs and associated signaling molecules concentrate, the peripheral SMAC (pSMAC), enriched in LFA-1 and other adhesion molecules, and the distal SMAC (dSMAC), where large surface receptors are confined and where an underlying ring of filamentous actin is localized to stabilize the global IS architecture (9). TCR engagement by the MHC-bound cognate ligand results in the activation of a branched signaling pathway that begins with the phosphorylation of the ITAM motifs in the subunits of the TCR-associated CD3 complexes by the Src family tyrosine kinases Lck and Fyn (10). Phosphorylated ITAMs become docking sites for the Syk family tyrosine kinase ZAP-70, that in turn recruits adaptor molecules to the nascent IS (10). The signaling modules fueled by early activation events, among which Ca^2+^-, diacylglycerol (DAG)- and phosphatidylinositol 4,5-bisphosphate (PIP_2_)- dependent signals, eventually converge to control cytoskeletal reorganization, a key pre-requisite for the formation of mature ISs (11). Translocation of the microtubule organizing center toward the T-cell/APC interface, which accompanies IS formation and accounts for the acquisition of a peculiar T cell polarity, allows for the directional release of vesicles containing effector molecules in the synaptic cleft (12, 13). Both TH and CTLs exploit vesicle release as a means to exert their effector functions. Different TH subtypes release subsets of cytokines which play highly specialized functions in both innate and adaptive immune cells, including fostering pathogen lysis and phagocytosis, tissue repair, release of anti-microbial peptides and germinal center formation (14). On the other hand, CTLs exploit IS formation to allow for polarized release of specialized secretory lysosome, called cytotoxic granules (CGs), characterized by a dense core of cytotoxic components, including the pore-forming protein perforin, proteases known as granzymes (GZMs), and the proteoglycan serglycin (15).

TCR signaling quickly propagates to ensure rapid responses to foreign antigens. However, after the response has been triggered, signaling must be equally rapidly turned off to discourage uncontrolled and potentially tissue-damaging T cell activation. Signal reversibility is guaranteed by efficient addition/removal of phosphate groups by ready-to-use phosphatases (16, 17). Among them SHP-1, a well-known mediator of TCR signaling termination, which mainly dephosphorylates Lck (18), and the non-receptor protein tyrosine phosphatases (PTPNs) -3, -4, and -22, which dephosphorylate both the ζ-chains of CD3 and ZAP-70 (19, 20). Enzymes that break down intracellular second messengers are also implicated in TCR signaling downregulation. This is exemplified by the inositol phosphatase SHIP-1, which dephosphorylates both PIP2 and PIP3 to decrease their local concentration (11, 21).

**Figure 1:**
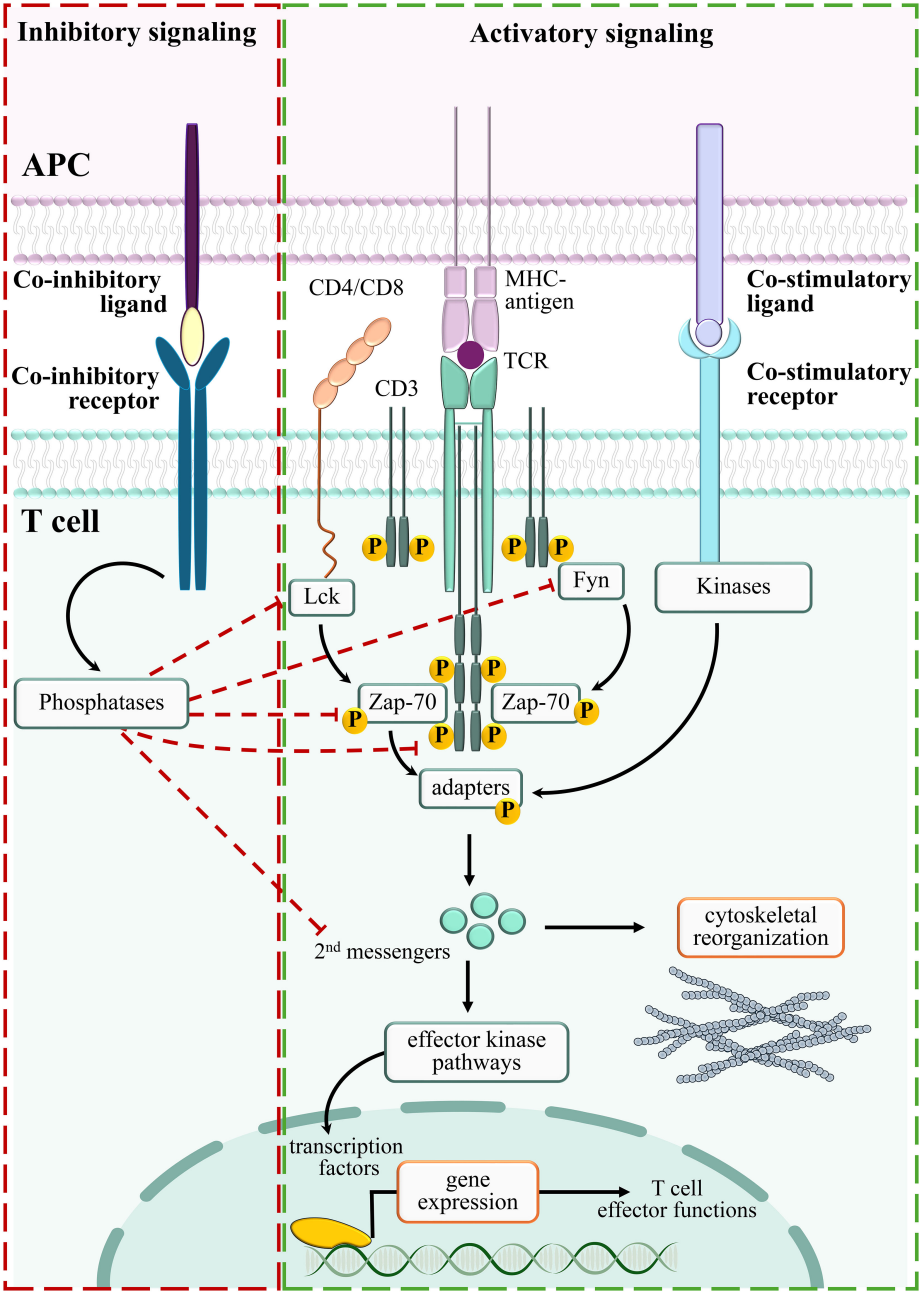
Positive and negative regulators of TCR signaling. Stimulation of TCR/CD3 complexes by MHC-bound antigens activates an integrated signaling pathway which delivers proliferation/differentiation/cytoskeleton reorganization signals (Activatory signaling, green box). By suppressing the TCR-dependent signaling pathways, negative regulators counteract T cell activation (Inhibitory signaling, red box).

Either lateral mobility along the plasma membrane or polarized vesicular trafficking deliver a large set of surface immune checkpoints to the IS, where they implement smart TCR signaling-interfering mechanisms (22, 23). By recruiting the tyrosine phosphatase SHP-2 to the immunoreceptor tyrosine-based switch motif (ITSM) localized in its cytoplasmic tail (24, 25), which becomes transiently phosphorylated following binding to its ligands Programmed death-ligand 1/2 (PD-L1 and PD-L2) expressed on APCs (26), the co-inhibitory checkpoint Programmed Death-1 (PD-1) is a paradigm of TCR signaling inhibitory molecules (11). The co-inhibitory checkpoint Cytotoxic T-lymphocyte-associated protein 4 (CTLA-4) binds to B7.1 and B7.2 and depletes them from neighboring cells by trans-endocytosis (27, 28). In mature IS, CTLA-4 localizes to the cSMAC where it interacts with SHP-1 (29) and with the serine/threonine phosphatase PP2A (30), reverting the phosphorylation of TCR signaling mediators (3, 31) and impairing IS assembly (22). The transmembrane molecule Lymphocyte-Activation Gene 3 (LAG-3) also inhibits TCR signaling (32) by associating with the TCR/CD3 complex and lowering cytoplasmic pH close to the IS, thereby causing the dissociation of Lck from the CD4 or CD8 co-receptors and hampering T cell activation (33, 34). To the group of co-inhibitory surface molecules also belongs T cell Immunoglobulin and Mucin domain-containing protein 3 (TIM-3) (35), which is recruited to the IS following T cell activation (36), where it binds the Lck-inhibitory kinase Csk, thereby suppressing antigen-dependent signaling (37) (**Figure 1**).

## 3 Soluble factors operate T cell suppression in the TME

The interplay between stimulatory and inhibitory molecules controls both the duration and outcome of the signaling cascades initiated by the TCR. This tight balance is significantly influenced by environmental factors, such as neighboring cells and soluble molecules, each of them contributing in its own way to ensure T cell activation, at the same time preventing aberrant T cell responses (38, 39). However, under certain conditions, environmental factors suppress effective T cell responses, contributing to disease onset or development. This “improper resource allocation” is implemented in the TME, a complex and multifaceted niche where tumor cells live and grow in close proximity to a plethora of other cells - fibroblasts, immune and inflammatory cells, stromal cells, endothelial cells - and in the presence of soluble molecules either released by resident/recruited cells or infiltrated from nearby areas (40).

A profound subversion of the balance between stimulatory and inhibitory molecules is at the basis of the defective activation of key TCR-dependent signaling molecules (41), altered IS architecture (42), and impaired effector T cell functions (43, 44) observed in the TME. In several types of human cancers including, among others, melanoma (45), ovarian cancer (46), non-small cell lung carcinoma (47), and hematologic malignancies such as lymphomas (48–50), recruited tumor-specific effector T cells, which acquire the definition of tumor-infiltrating lymphocytes (TILs), overexpress the exhaustion markers CTLA-4, TIM-3, LAG-3 and PD-1 (44, 51–53), which elicit immunosuppressive signaling cascades leading to T cell dysfunction.

**Figure 2:**
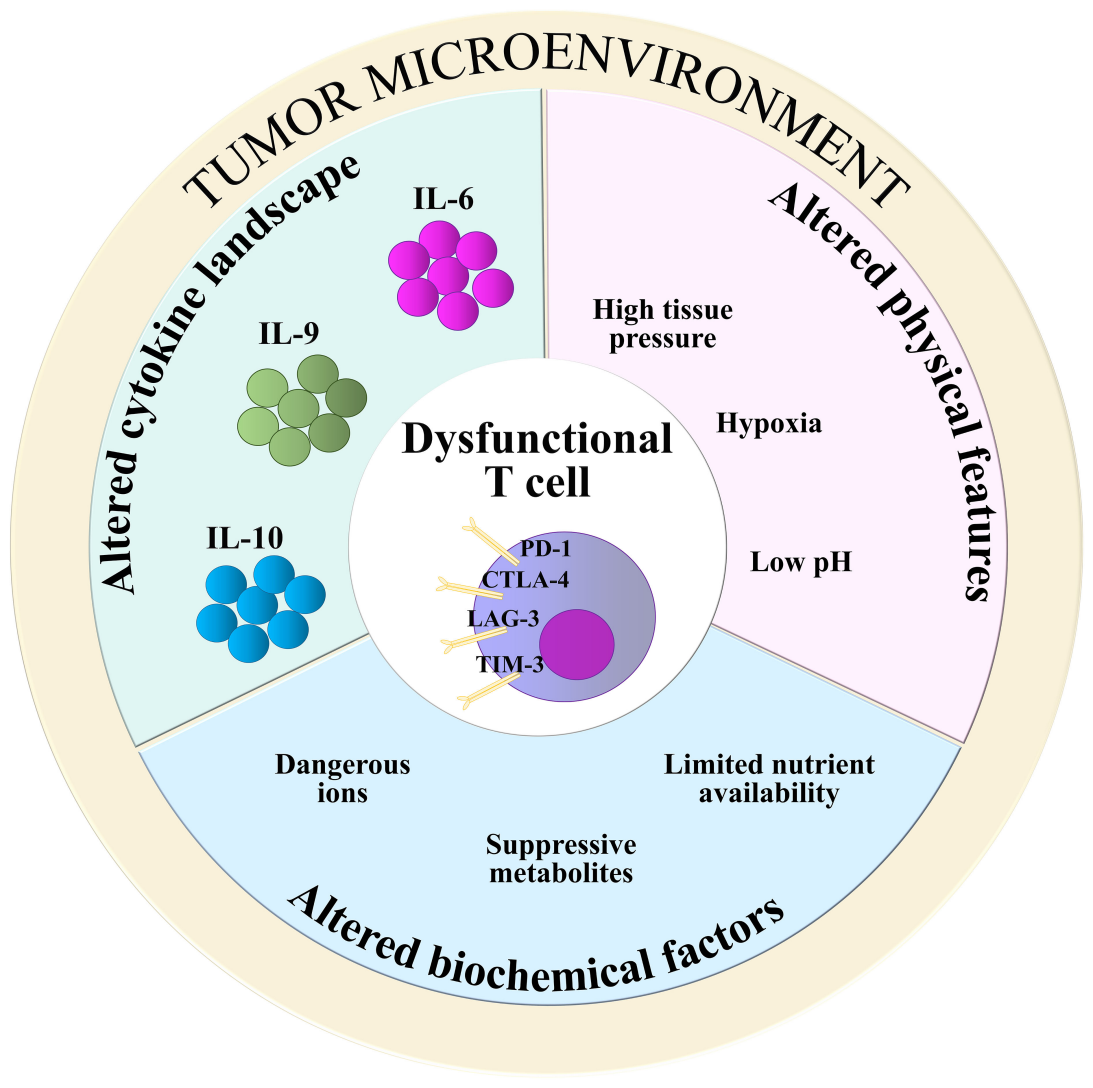
TME-derived T cell-suppressive factors. Alterations in biochemical composition (light blue), physical features (pink), and cytokine landscape (green) of the TME contribute to impair the anti-tumoral functions of T cells. Suppressive metabolites: fumarate, kynurenine, cholesterol, adenosine; Dangerous ions: [K^+^]; Nutrients: glucose, aminoacids.

Physical and chemical features of the TME, such as hypoxia, low pH and high tissue pressure, contribute to generate a tumor-promoting environment where TILs, notwithstanding their antigen specificity, are incapable to carry out efficient anti-tumor activity (8, 39). The release of dangerous catabolites by metabolically-rewired tumor cells, as well as competition for available glucose and aminoacids, also account for T cell suppression in the TME (39, 54). This is exemplified by the accumulation of potassium ions [K^+^], which suppress both TCR signaling and T cell effector functions by activating PP2A (55). Moreover, TME-populating cells other than tumor cells, such as tumor-associated macrophages, neutrophils, myeloid-derived suppressor cells, regulatory T cells (Tregs) and cancer-associated fibroblasts (CAFs) (7), compete with TILs for nutrients, at the same time producing immunosuppressive byproducts. Among them, fumarate has been recently found to impair the activity of ZAP-70 in CD8^+^ TILs, contributing to abrogate their anti-tumor activities (56). In M2 type macrophages, arginase-1 depletes L-arginine from the TME, while indoleamine-2,3-oxygenase (IDO) converts tryptophan to immunosuppressive kynurenine derivatives (57). Moreover, through the CD39/CD73-mediated catalysis of ATP, Tregs produce adenosine, a suppressive metabolite that binds to adenosine receptors (A2AR) on TILs and impairs NF-κB signaling (38, 58). Cholesterol, a metabolic byproduct of tumor cell metabolism, also contributes to T cell dysfunction by inducing stress of the endoplasmic reticulum (59). Hence, T cells subjected to the synergic actions of metabolic byproducts become unable to efficiently eradicate tumors (**Figure 2**).

## 4 The curious case of the cytokines: unveiling their impact on the TME

**Figure 3:**
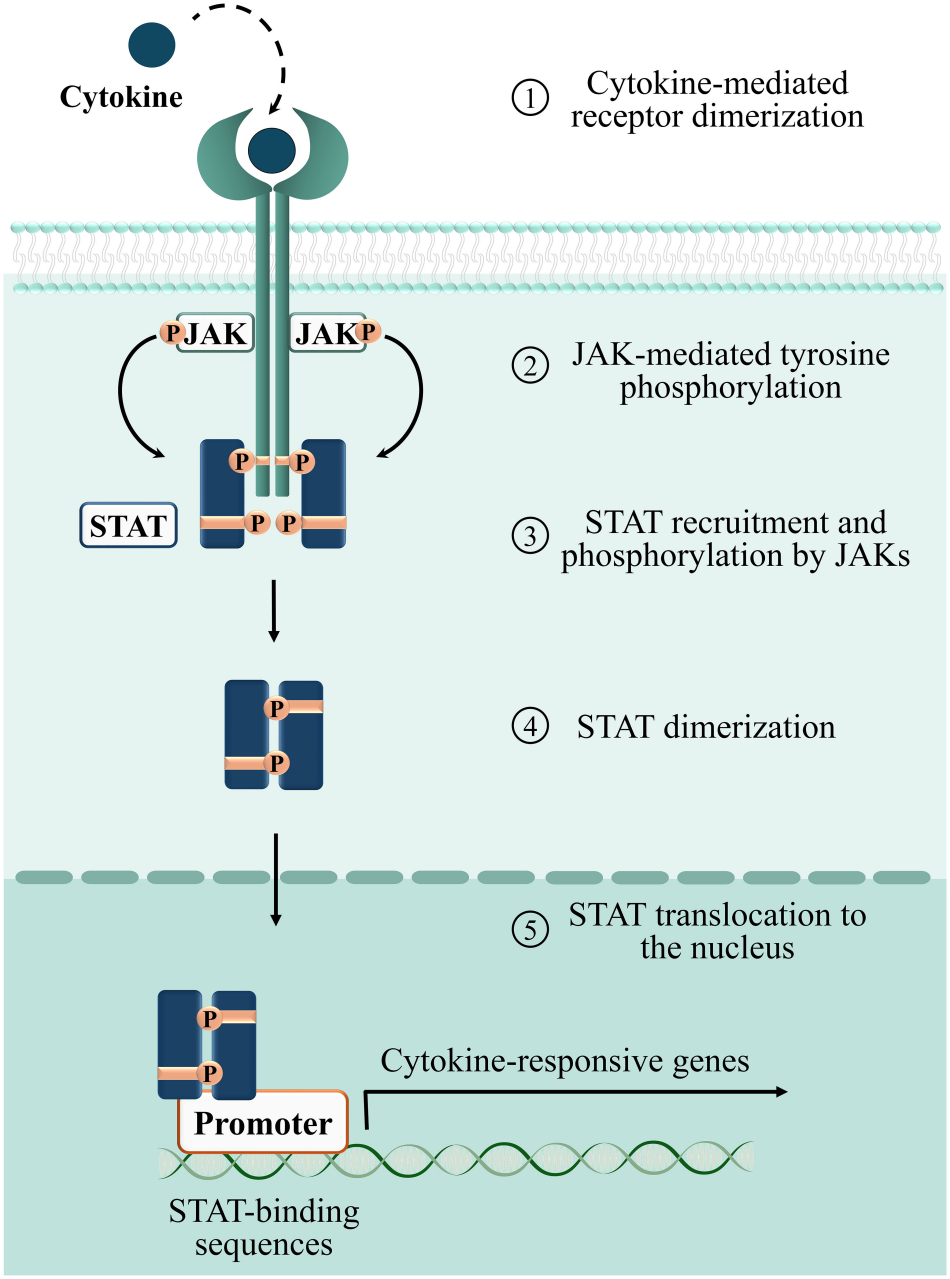
Phases of the cytokine-dependent JAK/STAT signaling cascade. 1: binding of cytokines to their corresponding surface receptors induces receptor dimerization, bringing two associated JAKs into proximity. 2: receptor bound JAKs undergo autophosphorylation and activation. Activated JAKs phosphorylate tyrosine residues on the receptor, providing docking sites for STATs. 3: STATs recruited to the phosphorylated receptor are phosphorylated and activated by JAKs. 4: phosphorylated STATs form homo/heterodimers. 5: STATs translocate to the nucleus and regulate transcription of target genes.

The large family of cytokines is subdivided in six groups: i) the IL‐1 family; ii) tumor necrosis factor (TNF) ‐α and related molecules; iii) the transforming growth factor (TGF) ‐β subgroup; iv) chemokines; v) cytokines that signal through receptor tyrosine kinases, and vi) cytokines that signal through the JAK/STAT signaling pathway (Morris, Kershaw, and Babon 2018). To the latter and largest group of cytokines (reviewed in (60)) belong both immunomodulatory and pro-inflammatory cytokines such as IL‐2 and interferon (IFN)-γ. To elicit specific responses in target cells, each member of this group activates a basic signaling module which only requires three components: receptor, intracellular kinases and transcription factors. The cytoplasmic tail of cytokine receptors, that lacks kinase activity, constitutively associates to tyrosine kinases belonging to the Janus Kinase (JAK) family (61, 62). Receptor association to the specific cytokine ligand leads to JAK trans-phosphorylation and activation, which in turn elicits receptor phosphorylation on specific tyrosine residues that become docking sites for transcription factors belonging to the Signal Transducers and Activators of Transcription (STAT) family. When recruited to the receptor, STATs are phosphorylated by JAKs, dissociate from the receptor and translocate to the nucleus to drive the expression of cytokine‐responsive genes (62, 63) (**Figure 3**).

Despite their common signal transduction modules, each cytokine harbors peculiar biological functions, that rely not only on the specific members of the JAK and STAT families that transduce its signaling pathway, but also on target cell sensitivity and environmental cues. Four members of the JAK family have been discovered to date, JAK1, JAK2, JAK3 and Tyrosine Kinase 2 (TYK2), with broad tissue expression (62). The STAT family is also a multicomponent family, which includes seven ubiquitously expressed members, STAT1, STAT2, STAT3, STAT4, STAT5a, STAT5b, and STAT6, which either homo- or hetero-dimerize according to the stimulus (63).

TME-populating immune and non-immune cells, and tumoral cells themselves, fuel neoplastic progression through the secretion of cytokines, that act as intercellular “info carriers” for a wide variety of processes ranging from hemopoiesis to inflammation and immune responses (60). It has been estimated that 10-25% of all cancers develop from chronic inflammatory diseases, that are characterized by a strong and prolonged dysregulation of pro-inflammatory cytokines (64, 65). Meta-analysis of more than 10,000 tumor samples collected within The Cancer Genome Atlas (TCGA) revealed that tumors can be grouped according to “immune subtypes”, defined not only by both extent and type of immune cell infiltration in the TME, but also by different mRNA expression profiles of cytokines and cytokine receptors (66, 67). Over the past 30 years it has become clear that malignant transformation causes a profound subversion of the cytokine landscape of the TME, where several cytokines, including IFN-γ (68), tumor necrosis factor (TNF)-α (69), TGF-β (70), IL-1 (71), IL-6 (72), IL-9 (73, 74), IL-10 (75), IL-15 (76), IL-27 (77), and IL-35 (78), abnormally released in the TME, acquire tumor-promoting activities. Most of them act by directly targeting tumor cells and activating STAT3-mediated signaling pathways which promote their survival (79–81). Cytokine-mediated signaling also enhances the surface expression of PD-L1 in tumor cells, thereby promoting their immunosuppressive potential (82). IFN-γ, a paradigmatic example of this circuitry, has been demonstrated to enhance PD-L1 expression in thirty-two tumor cell lines (82). Moreover, IL-27 stimulates Human Mesothelioma Cells to express and secrete PD-L1 (77). Interestingly, in the TME CD8^+^ T cells release high amounts of IFN-γ (83, 84), suggesting the existence of a immunosuppressive circuitry fueled by T cells themselves.

Of note, only a small subgroup of the above-mentioned cytokines, reviewed hereafter, demonstrated remarkable T cell-directed suppressive activities in the TME.

### 4.1 IL-6

**Figure 4:**
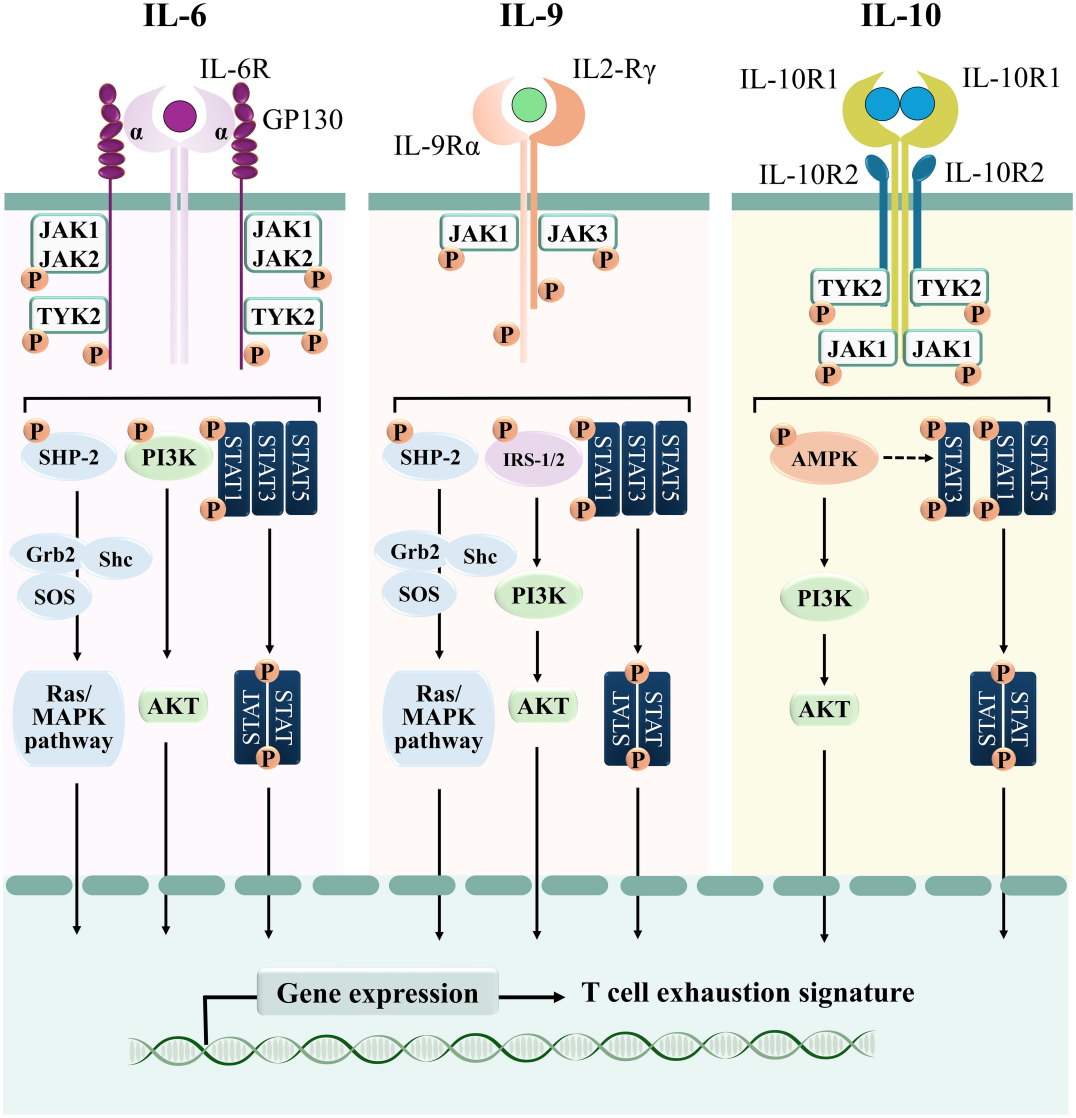
Signaling cascades of T cell-suppressive cytokines. Schematic representation of the signaling pathways elicited by IL-6, IL-9, and IL-10.

IL-6 is a pleiotropic cytokine whose molecular mass ranges from 21 to 28 kDa as a result of post-translational modifications (85, 86). It is secreted by immune and non-immune cells, among which fibroblasts, monocytes, mesangial cells, endothelial cells, keratinocytes, and T and B lymphocytes (87). The biological effects of IL-6 rely on its binding to the receptor Interleukin 6 Receptor (IL-6R), a complex consisting of an IL-6R alpha subunit (IL-6Rα, also known as CD126), that interacts with the ligand, and the IL-6 signal transducer Glycoprotein 130 (GP130). Binding of IL-6Rα to the cytokine leads to GP130 homodimerization (86), which rapidly activates JAK1, JAK2 and TYK2 to catalyze the phosphorylation of tyrosine residues within the cytoplasmic domain of GP130 (88, 89) (**Figure 4**). These sites act as docking regions for the STAT family transcription factors STAT1, STAT3 and, to a lesser extent, STAT5 (89). Interestingly, phosphorylated GP130 recruits and activates both the phosphatidylinositol 3-kinase (PI3K)/Ak strain transforming (AKT) pathway (90) and the SH2-domain Containing (SHC) adaptor, which in turn leads to the activation of members of the mitogen activated protein (MAP) kinase family (91). Phosphorylated GP130 also recruits SHP-2, which has been suggested to be involved in the termination of IL-6-mediated signaling (92) (**Figure 4**). While IL-6Rα expression is restricted to hepatocytes, megakaryocytes, and leukocytes such as monocytes, macrophages, and B and T lymphocytes (93, 94), GP130 is broadly expressed, with the notable exception of Tregs, which are therefore less sensitive to IL-6 with respect to the other T cell subsets (95).

IL-6 regulates a plethora of biological processes, including hematopoiesis, bone metabolism, embryonic development, and innate and adaptive immune responses, but most of all it is considered as a pro-inflammatory cytokine, as demonstrated by its abnormally high levels in sera of patients with chronic inflammatory diseases (93). Of note, it has been found to also accumulate in sera of patients with hematopoietic malignancies and solid tumors as a result of its secretion in the TME by various cell types, including cancer cells, CAFs and immune cells (87, 96). Clinical studies demonstrated that serum levels of IL-6 correlate with both stage and size of tumors, and predict both metastatic potential and patient survival (96). The pro-tumoral activities of IL-6 are related to its ability to directly act on tumor cells by activating STAT3, which in turn promotes their proliferation, survival, and invasive potential (72, 97). Interestingly, the STAT3-stimulating activities of IL-6 have been also recently associated to the suppression of T-cell anti-tumor activities (98). Increased IL-6 in sera of chronic lymphocytic leukemia (CLL) patients correlates with STAT3-dependent dampening of T cell proliferation and IL-2 production (99, 100). Huseni and colleagues recently reported that CD8^+^ T cells purified from peripheral blood of kidney and bladder cancer patients with high plasma IL-6 levels display a STAT3-dependent exhausted and repressed functional profile, with lower expression of genes involved in CTL activation, including CD28, and cell-to-cell cross-talk such as CD40L, and enhanced expression of the inhibitory receptors CTLA-4 and TIGIT, of the immunosuppressive cytokine IL-10 and of the transcription factors BATF, FOXO1, HIF-1α, and TOX2, which restrain effector differentiation of CD8^+^ T cells (101). The IL-6-STAT3 signaling axis also inhibits CTL differentiation *in vitro*, counteracting the TCR-dependent enhancement of GZMB, TNF-α and IFN-γ expression (101). Moreover, IL-6 released in the TME also suppresses the differentiation of IFN-γ-producing TH cells by attenuating both surface expression of MHC-II and secretion of IL-12 in dendritic cells of tumor-bearing mice, in a STAT3-dependent manner (102–104). On the other hand, *il-6* deletion in tumor-bearing mice promotes the anti-tumor activities of effector T cells and inhibits tumorigenesis *in vivo* (101, 104). Interestingly, in lung cancer, macrophage-derived IL-6 promotes CD8^+^ T cell exhaustion by enhancing the STAT3-dependent PD-1 expression (105, 106). This is in line with the decreased PD-1 expression and inhibited effector functions of T cells isolated from STAT3 conditional knock-out mice (107). These data provide evidence that IL-6, by activating STAT3, enhances the expression of PD-1, which in turn interferes with the TCR-dependent signaling pathway, thereby suppressing the anti-tumoral activities of T cells in the TME.

It is worth noting that, alongside its pro-tumoral functions, IL-6 has been observed to exert T cell-stimulating effects (108). Specifically, in T cells isolated from the peritoneum of mice exposed to pro-inflammatory stimuli, IL-6 promotes T cell proliferation (109) and induces the expression of the chemokine receptors CCR3, CCR4, CCR5, and CXCR3, which facilitate homing of T cells to lymphoid and non-lymphoid organs (110). IL-6 released by dendritic cells enhances activation, expansion, survival and polarization of T cells (108), while simultaneously inhibiting T cell apoptosis by upregulating the anti-apoptotic BCL-2 family proteins BCL-2 and BCL-2L1 (111). Moreover, the T cell-stimulating properties of IL-6 include inhibition of the immunosuppressive capacity of Tregs and interference with their differentiation from naïve T cells (112). Therefore, it is plausible that, within the intricate niche of the TME, the T cell-promoting effects of IL-6 may be overridden by the actions of other surface or soluble factors, resulting in T cell suppression. However, given the complexity of this topic, further investigations are needed to fully understand the impact of IL-6 on anti-tumoral T cell functions in specific pathological contexts.

### 4.2 IL-9

IL-9 is secreted by a number of cell subsets, including TH2 and TH17 cells, Tregs, the type 2 innate lymphoid cells (ILC2s), NK-T cells, and TH9 cells, a peculiar effector T cell population that differentiates starting from either naïve T cells in the presence of IL-1β, IL-4 and TGF-β, or from TH2 cells in the presence of TGF-β alone (74, 113). Recently, other cell populations have been found to secrete IL-9, among which TC9 (114), a modified CTL population that differentiates in an IL-9-enriched microenvironment and that expresses CTL-specific molecules, such as GZMB, the transcription factors EOMES and T-BET, and IFN-γ (74).

IL-9 is a member of the γ-chain family of cytokines. It binds to the heterodimeric surface receptor IL-9R, which is composed of a common γc chain and an IL-9Rα-specific chain that provides the ligand binding domain (115). IL-9 binding to the receptor induces its dimerization and cross-phosphorylation through both JAK1 and JAK3, which then promote recruitment and activation of STAT1, STAT3 and STAT5 (116) (**Figure 4**). Moreover, in hematopoietic cells IL-9 also activates the Insulin Receptor Substrates IRS-1 and IRS-2, large adaptor molecules that interact with the p85 regulatory subunit of PI3K, thereby activating downstream signaling molecules among which AKT, that in turn promotes T cell survival and prevents apoptosis (117). Weak activation of the MAPK pathway has been also reported in lymphoid and mast cell lines stimulated with IL-9, as a consequence of recruitment of the adaptors SHC and Growth factor receptor-bound protein 2 (GRB2) to the receptor (118) (**Figure 4**).

IL‐9Rα can be found at the surface of TH17 cells, Tregs, and CTLs and its binding to environmental IL-9 leads to a variety of cellular effects ranging from stimulation of TH17 differentiation and proliferation to enhancement of the suppressive activities of Tregs and modulation of CTL cytotoxicity (119). It is noteworthy that, in different disease contexts, this wide variety of effects differently impinge on T cell-mediated responses. In allergic diseases, IL-9 exerts a detrimental pro-inflammatory activity by promoting the expression of the chemokines CCL17 and CCL22 by TH2 lymphocytes (120). Moreover, overexpression of IL-9 and IL-9Rα in interstitial fluids and tissues isolated from patients with autoimmune diseases has been related to the degree of tissue inflammation (121, 122). On the other hand, IL-9 expression exerts an anti-inflammatory activity in some types of autoimmune diseases, such as multiple sclerosis, where it impairs the secretion of Granulocyte-Macrophage Colony-Stimulating Factor (GM-CSF) by CD4^+^ T cells, thereby reducing autoimmune neuroinflammation (123).

In the majority of solid tumors IL-9 acts as an anti-tumoral factor by promoting tumor cell apoptosis and activating innate and adaptive anti-tumoral immune responses (124, 125). In mouse models of melanoma, injection of recombinant IL-9 inhibits tumor growth (126). Moreover, in mouse models of melanoma and breast cancer IL-9 released in the TME forces macrophages to release the chemokines MIP-1 and CXCR3, which in turn attract T cells, thereby enhancing anti-tumor immune responses (127). In gastric cancer patients, its high intra-tumoral expression has been associated with increased numbers and elevated killing activities of CD8^+^ TILs, enhanced efficacy of anti-PD-1 immunotherapy based on the monoclonal antibody Pembrolizumab, and increased overall survival (128). Colon cancer patients show a strong correlation between IL-9 expression and disease progression, with the better prognosis shown by patients with the highest levels of IL-9 in cancer tissues (129). Furthermore, increased IL-9 concentrations in peripheral blood of breast cancer patients promote cytotoxicity of tumor-specific CTLs (130). In a mouse model of colorectal cancer, IL-9 suppressed tumor growth through CD8^+^ T cell activation (131). Similar findings were observed in mouse melanoma models, where *in vivo* IL-9 blockade promoted melanoma progression, while on the other hand recombinant IL-9 enhanced the cytotoxic ability of murine melanoma-specific CD8^+^ T cells (132). Therefore, we can hypothesize that the role of IL-9 in hampering the anti-tumoral activities of T cells depends on the specific cancer type (133).

As opposed to solid tumors, IL-9 mainly exerts a pro-tumoral effect in B and T cell hematologic malignancies, which results from its ability to trigger the JAK/STAT pathways, that eventually stimulate neoplastic cell accumulation and promote disease progression (125, 134). Enhanced expression of IL-9 and IL-9Rα is detectable in biopsies and sera from patients with several hematologic neoplasias and correlates with adverse prognostic markers of the diseases (extensively reviewed in (122)). Neoplastic cells from T-ALL and Cutaneous T-Cell Lymphoma patients are highly sensitive to IL-9, which promotes Ras/MAPK signaling and enhances their proliferation and survival (135, 136). Upregulation of IL-9 levels in the plasma of multiple myeloma patients correlates with disease severity (137). In the context of CLL, high amounts of IL-9 secreted by TH9 and leukemic cells correlate with hallmarks of aggressive disease and lower overall survival (74). IL-9 released at abnormally high levels in the TME of CLL acts on stromal cells by enhancing their secretion of homing chemokines, which in turn favor leukemic cell accumulation in the pro-survival and chemoprotective lymphoid niche (74, 122).

Very limited information is available concerning the ability of IL-9 to interfere with the anti-tumor functions of T cells. In mouse models of non-small cell lung cancer, IL-9 demonstrated a CD8^+^ T cell-suppressive activity, which was mediated by its ability to enhance PD-1 expression, an effect which was neutralized by PD-1-neutralizing antibodies (138). In these disease models, IL-9 was found to target both CD4^+^ and CD8^+^ TILs, suppressing their secretory ability and impairing their anti-tumoral functions (139). We have recently dissected the molecular mechanism underlying the T cell-suppressive function of IL-9 in CLL. We reported that IL-9, aberrantly secreted by leukemic cells isolated from peripheral blood of CLL patients, promotes PD-1 expression in CTLs, thereby strongly affecting their ability to form productive ISs and to kill target cells (44). Altogether, these results suggest that IL-9 exerts opposite effects on T cell suppression according to the type of neoplastic cell and to its microenvironmental niche (113).

### 4.3 IL-10

In its biologically active form, interleukin-10 (IL-10) exists as a homodimer that binds to the tetrameric heterodimer IL-10 receptor (IL-10R) (140). Upon ligand binding, IL-10R promotes the phosphorylation of JAK1 and TYK2, allowing the recruitment of STAT1, STAT3, and STAT5 (8). Of note, while STAT1 and STAT5 are activated through the “classical” JAK/STAT module, IL-10-dependent STAT3 activation seems to be mainly mediated by AMP-activated protein kinase (AMPK) (141). In addition to STAT3, AMPK also promotes the activation of the PI3K/Akt signaling cascade in macrophages (142) (**Figure 4**).

Secreted by several immune cell types, including activated T cells, and mostly Tregs, monocytes, macrophages, dendritic cells, NK cells and B cells, IL-10 mainly targets immune cells themselves, resulting in a broad range of anti-inflammatory and immunosuppressive activities associated with the resolution of inflammatory diseases (75, 143, 144). These activities rely on the ability of IL-10 to inhibit the secretion of i) IFN-γ and IL-2 by TH1 lymphocytes, ii) IL-4 and IL-5 by TH2 lymphocytes, iii) IL-1β, IL-6, IL-8, IL-12, and TNF-α by phagocytes, and iv) IFN-γ and TNF-α by NK cells (145). The suppressive functions of IL-10 on TH cells have been ascribed to its ability to suppress the expression of the co-stimulatory molecules CD28 and ICOS, thereby interfering with cytokine production (146). IL-10 also indirectly suppresses TH cell functions by interfering with the expression of MHC-II and of the co-stimulatory molecules B7.1/2 by APCs, thereby impairing their ability to provide the accessory signals necessary for TH cell activation (147).

The anti-inflammatory functions of IL-10 initially fueled the idea that it acts as a suppressor of immune responses in the tumor context, thereby favoring tumor development. This idea was corroborated by the finding that IL-10 promotes both survival and proliferation of tumor cells by stimulating STAT3 activation, at the same time hampering tumor antigen presentation to immune cells, which enhances evasion of immune surveillance (148). High amounts of IL-10 in sera of diffuse large B-cell lymphoma (DLBCL) (80), CLL (149), glioma (150), and cutaneous T cell lymphoma (151) patients are considered as a marker of unfavorable prognosis. DLBCL cells express both IL-10 and its receptor, eliciting an autocrine pathway of STAT3 activation that fuels tumor cell proliferation (80). Recently, IL-10 has been also implicated in shifting macrophage polarization toward the tumor-promoting M2-like phenotype, that in turn secretes TGF-β and IL-6 (7, 152, 153). Moreover, high degrees of IL-10^+^ TAM infiltrates associate with CD8^+^ T cells exhaustion and worst prognosis in muscle-invasive bladder cancer patients (154). This concept is corroborated by the ability of IL-10 to promote both differentiation and suppressive functions of Tregs, which in turn secrete large amounts of IL-10 in the TME (155). This positive feedback loop supports the establishment of an immunosuppressive microenvironment that favors tumor growth and progression (155) by enhancing the expression of PD-1 and promoting the transcriptomic signature of exhaustion in CD8^+^ TILs (156).

Of note, the outcomes of IL-10 depend on both cell types and environmental conditions. Indeed, in B cells it stimulates proliferation and promotes immunoglobulin secretion and isotype switch (145, 157). IL-10 also induces activatory signals in CD8^+^ T cells, where it promotes target cell killing during acute infection (158). Hence, IL-10 enhances both cytotoxic and humoral responses against pathogens, at the same time suppressing the helper activities of CD4^+^ T cells.

In the TME, where chronic inflammation fuels tumor growth, the anti-inflammatory activities of IL-10 have been implicated in harnessing the anti-tumoral functions of CD8^+^ T cells (8, 159). In mice with colorectal cancer cell-derived liver metastases, IL-10 upregulates PD-L1 expression in monocytes, which in turn reduces CD8^+^ T cell infiltration and anti-tumor immunity (160). In CLL and breast cancer patients, reduced IL-10 expression or impaired IL-10R-STAT3 signaling is correlated with increased frequencies of exhausted CD8^+^ T cells (161). Sun and colleagues demonstrated that STAT3, activated by IL-10 released in the TME, enhances the anti-tumoral activities of CTLs and promotes their differentiation in mouse models of melanoma (162). Moreover, mice and humans deficient in IL-10 signaling components spontaneously develop tumors. Interestingly, TCR stimulation in CD8^+^ T cells leads to upregulated expression of IL-10R, thereby enhancing both IL-10 sensitivity and IL-10-mediated STAT3 activation, which have been both related to enhanced CD8^+^ T cell survival (148, 163). Collectively, these data provide evidence that cancer type-specific TME-associated factors govern the ability of IL-10 to act as either a pro- or an anti-tumoral cytokine, and highlight the need for further studies to clarify the functions of IL-10 in specific pathological contexts.

## 5 Rewiring the cytokine landscape of the TME as new pharmacological anti-cancer tool

Chemotherapy and radiation therapy have been the mainstay of cancer treatment for decades, notwithstanding their significant side effects and their uncertain benefits in terms of cancer growth control (164). In recent years several approaches have been applied to implement traditional clinical practice by targeting cancer cells at the same time leaving untouched normal cells. After the discovery of immune checkpoint expression and functions, pharmacological targeting of immune checkpoints seemed a viable option to counteract T cell suppression in cancer. Over the past decade immune checkpoint-neutralizing antibodies, now widely known as “immune checkpoint inhibitors”, showed promising results in clinical trials as promoters of T cell anti-tumoral functions, and were approved by the US Food and Drug Administration (FDA) to treat some cancer types, including melanoma and bladder and lung cancers (165). However, notwithstanding their clear-cut therapeutic success, a high percentage of tumors remain innately resistant to immune checkpoint inhibitors, or become resistant throughout treatment programs (166).

CAR T-cell therapy, which involves extracting T cells from a patient’s blood, genetically modifying them to recognize and attack tumor cells, and reintroducing them into the patient’s body, also falls under the classification of immunotherapy. Engineered T cells acquire tumor-specific features that enable them to localize and eliminate tumor cells. Unfortunately, the therapeutical success of CAR T cell-based therapies for the treatment of some hematological neoplasms such as B-acute lymphoblastic leukemia (ALL) and large B cell lymphoma, are challenging to replicate in other tumoral contexts, particularly solid tumors (167). Although the exact reasons for the therapeutic failure of CAR T cells remain to be fully elucidated, the mechanisms employed by the TME to suppress the anti-tumoral activities of CTLs may also contribute to the suppression of CAR T cells. A comprehensive understanding of the mechanisms utilized by the TME to inhibit CTL (and CAR T cell) anti-tumoral activity is therefore necessary to expand our treatment options.

**Figure 5:**
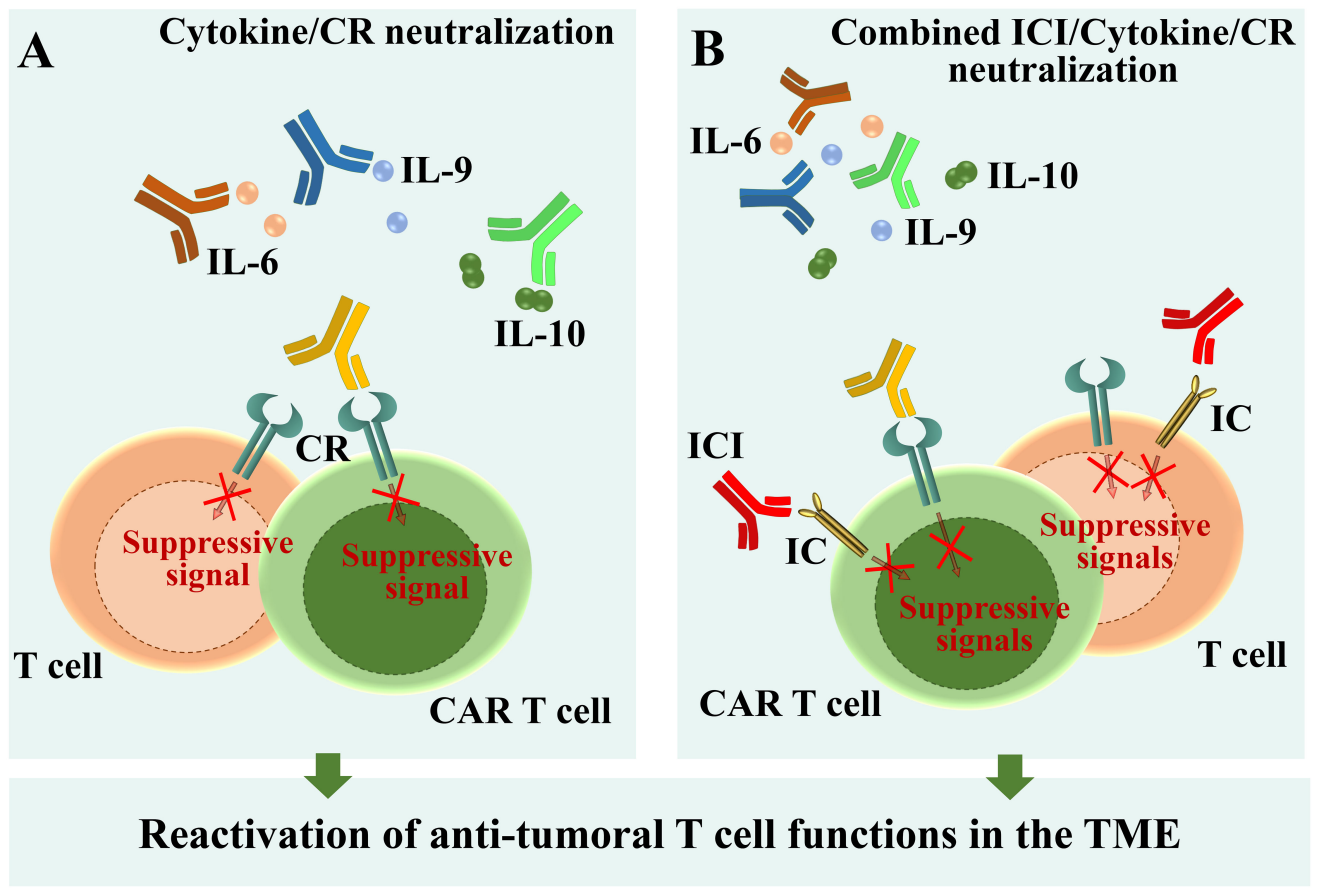
Schematic overview of available therapies and new perspectives for T cell reactivation. **(A)** Neutralization of immunosuppressive cytokines by specific antibodies. **(B)** Combined immune checkpoint inhibition and cytokine/cytokine receptor neutralization through specific antibodies. ICI, Immune checkpoint inhibitor; CR, Cytokine receptor.

In the last decade, cytokines which appeared to impair the anti-tumoral functions of T cells have been explored as potential targets for immunotherapy. Large clinical trials demonstrated that high amounts of IL-6 found in sera of advanced kidney, breast, and hepatocellular carcinoma, non-small cell lung cancer and bladder cancer patients correlate with poor response to the anti-PD-L1 antibody atezolizumab (101, 168–170). *In vivo* administration of monoclonal anti-IL-6R neutralizing antibodies in tumor-bearing mouse models enhanced T cell responses and inhibited tumor growth (103, 104) (**Figure 5A**). Moreover, combined PD-L1 and IL-6R blockade caused tumor regression in pre-clinical models of solid tumors, improving CTL anti-tumor responses compared with anti-PD-L1 alone (101) (**Figure 5B**). In a recent phase I clinical trial using the anti-IL-6R mAb tocilizumab in combination with carboplatin/doxorubicin conducted in patients with recurrent epithelial ovarian cancer, T cells of IL-6R mAb-treated patients exhibited features of enhanced activation and secreted high amounts of effector cytokines (171). These successful experiments supported the idea that IL-6/IL-6R neutralization may by exploited to reactivate the T cell compartment and counteract tumor development. Clinical trials combining tocilizumab with either carboplatin (ClinicalTrials.gov Identifier: NCT05846789), or with atezolizumab (ClinicalTrials.gov Identifier: NCT04691817) are currently recruiting tumor patients refractory to first-line line immune checkpoint inhibitor-based therapies. Hopefully, these trials will soon provide insights into the efficacy and safety of IL-6/IL-6R neutralization as a therapeutic strategy for reactivating the T cell compartment and combating tumor development in patients refractory to first-line immune checkpoint inhibitor-based therapies (**Figure 5A, B**).

The anti-tumor efficacy of IL-9 neutralization has been also recently explored. Encouraging results, obtained by administrating anti-IL-9 antibodies in Eμ-TCL1 mice, the mouse model of CLL (122), demonstrate that this approach counteracts leukemia progression by on the one hand decreasing PD-1 expression in CD8^+^ T cells (44) and on the other hand lowering the expression of homing chemokines by stromal cells of lymphoid organs (74). Hence, IL-9 neutralization counteracts IL-9-dependent T cell exhaustion, at the same time preventing leukemic cell homing to the pro-survival niche of lymphoid organs. Moreover, neutralizing anti-IL-9 or anti-IL-9R antibodies significantly inhibit tumor growth in mouse models of lymphoma (172). These data open to the possibility of exploring IL-9 and IL-9R neutralization as a new anti-tumor approach (**Figure 5A**).

Combined treatments targeting either Tregs or IL-10 along with the PD-1 checkpoint pathway reversed T cell exhaustion during viral infections (173, 174), suggesting that similar pharmacological approaches might be applicable also to the tumoral context. Indeed, using organotypic cell culture approaches, Sullivan and colleagues found that IL-10 blockade potentiates anti-tumor T cell responses and decreases the percentage of exhausted CD8^+^ T cells in human colorectal cancer liver metastases. Interestingly, IL-10 neutralization also rescued both proliferation and cytotoxicity of tumor-specific CAR T cells (175). Moreover, IL-10 suppression enhanced both anti-tumor activity and responses to checkpoint blockade in CLL (176, 177) (**Figure 5B**). Therefore, neutralizing the effects of IL-10 holds therapeutic potential not only as a stand-alone treatment but also to enhance the function of adoptively transferred CAR T cells (**Figure 5A**). The ability of IL-10, but not of IL-6, to fuel the immunosuppressive functions of Tregs (112, 155), makes IL-10 neutralization in tumoral contexts even more promising.

As mentioned above, IL-10 also exerts anti-tumoral activities, which are being exploited as potential therapeutical strategy. Both IL-10 overexpression and treatment with pegylated IL-10 (PEG-IL-10), lead to enhanced expression of IFN-γ and GZMs in tumor-resident CD8^+^ T cells, thereby promoting their long-lasting cytotoxic ability and favoring tumor rejection in a subset of patients with intermediate to poor risk in renal cell cancer (163). More recently, mouse models of solid tumors treated with a IL-10/Fc fusion protein showed expansion and increased effector functions of exhausted CD8^+^ TILs, which resulted in tumor eradication (178). Zhao and colleagues engineered CAR T cells to secrete IL-10, and tested them in syngeneic and xenograft mouse models of colon cancer, breast cancer, pancreatic cancer and melanoma, where IL-10 secretion promoted both proliferation and effector functions of CAR T cells, leading to complete regression of established solid tumors and metastatic cancers (179). These findings highlight IL-10 as a key player in anti-tumor immunity and provide a strong rationale for further exploration of IL-10-based therapies in clinical settings.

Molecular components of the JAK/STAT signal transduction pathways are increasingly recognized as potential therapeutic targets against tumors (63). Cytokine-dependent alterations in these pathways have been associated with various cancers, such as cutaneous T-cell lymphoma, lung cancer, gastric cancer and colon cancer (180, 181). Notably, aberrant activation of STAT3, as described earlier, is widely implicated in cancer cell survival, immunosuppression and persistent inflammation within the TME (182). Targeting JAK and STAT proteins with specific inhibitors, either alone or in combination with anti-PD-1/PD-L1 neutralizing antibodies, represents a promising emerging therapeutic approach against cancer. The strong potential of these classes of inhibitors was demonstrated in preclinical models of colorectal cancer treated *in vivo* with nanoparticles loaded with the STAT3 inhibitor BBI608, which resulted in enhanced infiltration of CD4^+^ and CD8^+^ TILs at tumor sites and tumor regression (183). The development of JAK/STAT inhibitors has progressed rapidly, with several molecules currently under evaluation in clinical trials for cancer treatment. Among them, the JAK1/2 inhibitor Ruxolitinib has demonstrated remarkable anti-cancer effects, alone or in combination with the anti-PD-1 antibody nivolumab, in patients with multiple myeloma and Hodgkin lymphoma, including enhancing the cytotoxic capacity of T cells against tumor cells (184, 185). Clinical trials are currently underway to investigate the effects of the STAT3 inhibitor Napabucasin alone or in combination with PD-1 neutralizing antibodies in metastatic colorectal cancer (ClinicalTrials.gov Identifiers: NCT03522649, NCT02753127). Notwithstanding the large number of existing data linking these drugs to impaired T cell responses (186), results obtained from these recent clinical studies might provide valuable insights into the therapeutic potential of JAK/STAT inhibitors and their combination strategies in improving outcomes for cancer patients.

In addition to classical pharmacological approaches, gene engineering techniques are being explored as a means to interfere with cytokine-dependent signaling and reactivate T lymphocytes and CAR T cells against cancer. T cells expressing a chimeric receptor containing the extracellular domain of IL-2R fused with the intracellular domain of IL-9R acquire features of effector T cells and show anti-tumor activity when adoptively transferred in syngeneic mouse models of melanoma and pancreatic cancer (187). Liang and colleagues co-expressed SMAD7, an inhibitor of signaling pathways elicited by the immunosuppressive cytokine TGF-β (188), with a human epidermal receptor (HER) 2-targeted CAR in engineered T cells. SMAD7-engineered CAR T cells showed decreased exhaustion *in vitro* and demonstrated enhanced anti-tumoral effects in mouse xenograft models of solid tumors (189). We can hypothesize that a similar approach may be applicable to the T cell-suppressive JAK/STAT-dependent signaling pathways elicited by the cytokines described above, making it suitable for the treatment of tumors with peculiar cytokine landscapes of the TME. However, the large overlap of cytokine-dependent signaling pathways makes these pharmacological approaches potentially detrimental, suggesting that additional investigation is required to avoid undesired side-effects.

As extensively reviewed in (190), the intrinsic 3D structure of hematologic neoplasms, where tumor cells and T cells are in close physical contact, substantiates immunosuppressive cytokine neutralization and/or signaling cascade inhibition as efficient strategies to reactivate anti-tumoral T cell functions. However, solid tumor tissues usually impair T cell infiltration in the TME, thereby potentially frustrating the implementation of these therapeutic approaches. New microphysiological systems are currently under development for the evaluation of drugs that counteract T cell exclusion in the TME of solid tumors (190). These drugs, in combination with therapies aimed at neutralizing the T cell suppressive activities of IL-6, IL-9 and/or IL-10, may reasonably help reducing immune evasion of tumor cells, thereby ameliorating the clinical management of the disease.

## 6 Conclusions

Rewiring the TME to a non-permissive environment for tumor growth has emerged as a significant research challenge. However, notwithstanding remarkable breakthroughs obtained by applying precision medicine, nanomedicine and immunotherapy to multiple tumor types (191), we are still far from achieving complete success (192). Profound differences among tumor cells, as well as among their tissue of origin, generate tumor niches of peculiar compositions, where a multitude of cells interact in complex and sometimes unpredictable ways. Additionally, the landscape of released cytokines varies considerably across different TMEs, exerting diverse and occasionally opposite effects on their target cells.

A common observation across most tumor niches is the suppression of T cell-mediated anti-tumor responses. In this comprehensive review, we elucidated the current understanding of the mechanisms orchestrated by IL-6, IL-9, and IL-10, of which high amounts have been found within the TME of specific tumors, effectively hindering the anti-tumoral functions of T cells. Furthermore, we explore the contrasting roles of these cytokines in various tumoral contexts, highlighting the dynamic nature of the cytokine expression profile within the TME. This profile not only varies from one tumor to another but also exhibits inter-patient heterogeneity, suggesting a significant contribution to immunosuppression in a tumor-selective manner.

In this scenario, unveiling the cytokine landscape of each tumor type becomes critical for identifying and characterizing soluble factors that suppress of CTL-mediated killing. Moreover, understanding these factors would shed light on their potential implications in the poor response to CAR T cell therapies, facilitating the development of more effective treatments and improving outcomes for cancer patients.
